# Interstitial lung diseases (ILD) in common variable immunodeficiency (CVID) patients: a study from Iran

**DOI:** 10.1186/s12865-024-00640-0

**Published:** 2024-07-16

**Authors:** Ghamartaj Khanbabaee, Fatemeh khazaii, Zahra Chavoshzadeh, Mahsa Rekabi, Zahra Ghomi, Vahide Zeinali, Matin Pourghasem, Maedeh Soflaee, Mahsa Ghadrdan

**Affiliations:** 1 Department of Pediatric Pulmonology, Mofid Children’s Hospital, Shahid Beheshti University of Medical Sciences, Tehran, Iran; 2 Department of Immunology and allergy, Mofid Children’s Hospital, Shahid Beheshti University of Medical Sciences, Tehran, Iran; 3 Department of Immunology and allergy, Masih daneshvari Hospital, Shahid beheshti university of medical sciences, Tehran, Iran; 4 Department of clinical radiology, Mofid Children’s Hospital, ShahidBeheshti University of Medical Sciences, Tehran, Iran; 5 Research Institute for Children’s Health, Shahid Beheshti University of Medical Sciences, Tehran, Iran

**Keywords:** Primary immunodeficiency, Common variable immunodeficiency (CVID), Interstitial lung disease (ILD)

## Abstract

**Introduction:**

Interstitial lung disease (ILD) is a prevalent complication in patients with common variable immunodeficiency (CVID) and is often related to other characteristics such as bronchiectasis and autoimmunity. Because the term ILD encompasses a variety of acute and chronic pulmonary conditions, diagnosis is usually based on imaging features. Histopathology is less available. This study was conducted with the aim of investigating the ILD in patients with CVID.

**Materials and methods:**

In this retrospective cross-sectional study, sixty CVID patients who referred to the pulmonology and immunodeficiency clinics of Mofid Children’s Hospital between 2013 and 2022 were included. The diagnosis of ILD were based on transbronchial lung biopsy (TBB) or clinical and radiological symptoms. The prevalence of ILD in CVID patients was determined. Also, the CVID patients with and without ILD were compared in terms of demographic characteristics, clinical, laboratory and radiologic findings.

**Results:**

Among all patients, ten patients had ILD (16.6%). In terms of laboratory parameters, there was a significant difference between platelets in the two groups of CVID patients with and without ILD, and the level of platelets was higher in the group of patients with ILD. Moreover, in terms of clinical symptoms, pneumonia, diarrhea and hepatomegaly were significantly different between the two groups and were statistically higher in the group of patients with ILD (*P* < 0.05). Autoimmunity and malignancy were not significantly different in two groups. There was a significant difference in, hyperinflation between the two groups of CVID patients with and without ILD, and the frequency of, hyperinflation was higher in the patients without ILD (*P* = 0.040).

**Conclusion:**

Understanding the pathogenesis of ILD plays an essential role in revealing non-infectious pulmonary complications that occur in CVID patients. Increasing efforts to understand ILD not only shed light on its hidden pathogenesis and clinical features, but also enhance our understanding of CVID in a broader sense.

## Introduction

Common variable immunodeficiency (CVID) is the most prevalent form of clinically recognized primary immunodeficiency disease (PID), The diagnosis of CVID was made according to the updated diagnostic criteria of the European Society for Immunodeficiencies (ESID). Patients were required to be older than 4 years at the time of diagnosis to exclude transient hypogammaglobulinemia of infancy [1–3]. CVID disease can occur at any age, and most generally in individuals in their twenties or thirties, moreover the condition occurs equally in males and females [4, 5]. The most important clinical characteristic of CVID disease is frequent bacterial infection of the upper and/or lower respiratory tract [6, 7]. CVID can be classified into two major clinical phenotypes. One group experiences infection as the only major clinical manifestation, while the other group exhibits a variety of lymphoproliferative, inflammatory, and/or autoimmune complications [8].

Interstitial lung disease (ILD) is a term that includes a group of different acute and chronic lung conditions with common clinical and physiological characteristics [9]. This condition is almost a prevalent complication in CVID patients [10]. CVID patients with ILD often have a much more complicated course, and ILD increases morbidity and mortality in these patients [11].

The pathogenesis of CVID-associated ILD is considered to be independent of bacterial infection, as ILD may develop in the absence of bronchiectasis, and the presence of ILD is not related to the history of pneumonia [12, 13]. CVID patients with ILD show a different immunological and clinical phenotype compared to patients without ILD. Importantly, other complications associated with CVID, such as splenomegaly, autoimmune cytopenia, persistent lymphadenopathy, and lymphoproliferative were more frequent in the ILD patients [14, 15]. The diagnosis of ILD is usually based on clinical presentation and radiological features. For patients in whom a biopsy has been performed, histology provides further confirmation of the diagnosis along with individual pathologic features [10, 16]. Previous investigations have addressed the percentage of clinically diagnosed ILD in CVID, which has been reported in the range of 10–20% [17–19].

No previous study has evaluated the ILD in CVID patients in Iran, here we report the first Iranian cohort study of ILD in CVID patients in order to further elucidate the complex aspects of this identified disorder.

## Methods and materials

In this retrospective cross-sectional study, sixty CVID patients who referred to the pulmonology and immunodeficiency clinics of Mofid Children’s Hospital between 2013 and 2022 were enrolled. According to the Declaration of Helsinki, information related to the research was collected after obtaining informed consent from the patients.

Diagnostic Criteria The diagnosis of CVID was made according to the updated diagnostic criteria of the European Society for Immunodeficiencies (ESID). The criteria include markedly reduced serum levels of IgG (at least 2 SD below the mean for age) AND low levels of IgA AND/OR IgM, along with poor or absent response to vaccines, and exclusion of other causes of hypogammaglobulinemia. Patients were required to be older than 4 years at the time of diagnosis to exclude transient hypogammaglobulinemia of infancy [20]. Patients with heart diseases, hyperthyroidism, patients with other lung diseases for any reason except immunodeficiency, and patients under 4 years without continuous follow-up were excluded. In this study, the diagnosis of ILD was based on transbronchial biopsy (TBB) for two patients and on clinical and radiological findings includes “soft” centrilobular nodules, ground-glass opacification, smooth thickening of the interlobular septa, and lobular foci of decreased attenuation in HRCT scan, hypoxia, shortness of breath in clinical reviews for 8 patients (histopathology was less available). Then, the incidence of ILD in CVID patients was determined. Based on the eligibility criteria, 60 patients were divided into two groups: patients without ILD (*n* = 50), and patients with ILD (*n* = 10). A two-page questionnaire was designed to retrospectively collect all demographic, clinical, and laboratory information from the patients’ medical records. Demographic characteristics, clinical symptoms, and laboratory findings were compared in patients with and without ILD. Among 60 CVID patients, 2 subjects had no lung CT-scan report, 29 patients had lung involvement, and other patients had normal report. Pattern of lung involvement (*n* = 29) compared in CVID patients with and without ILD.

### Ethical consideration

The Ethics Committee of the Shahid Beheshti University of Medical Science approved this study (IR.SBMU.MSP.REC.1401.509). In addition, According to the Declaration of Helsinki, information related to the research was collected after obtaining informed consent from the patients.

### Statistical analysis

Statistical analysis was done using SPSS software version 22. The normal distribution of the data was evaluated using the Kolmogorov Smirnov test. Central and descriptive statistics were reported for quantitative data. For variables with skewed distribution, median and interquartile range (IQR) were used to describe quantitative variables, and frequency and percentage were used to describe qualitative variables. To compare quantitative and qualitative variables between two groups with and without ILD, Mann-Whitney test and Fisher’s exact test were used, respectively. In addition, Spearman test was used to measure the correlations. A two-tailed p-value less than 0.05 was considered significant.

## Results

Out of 60 patients with CVID, ten patients had ILD (16.6%). 58.4% of the participants (35 patients) were female. Twenty-two (40%) of the patients were born to consanguineous families. Gender was significantly different between CVID patients with and without ILD, and in the ILD group, most patients were male (*P* = 0.046). The positive family history of primary immunodeficiency was observed in 5 (8.3%) patients. The median (IQR) age at the time of the study was 27.0 (10.75–37.5) years for patients with ILD, and 17.0 (12.0–37.0) years for those without (*P* = 0.576). The median age at the onset of CVID symptoms was 6.0 years (IQR: 2.75-11.0), while the median age at the time of CVID diagnosis was 11.0 years (IQR: 4.0–21.0). The median delay in CVID diagnosis was 3.9 years (IQR: 0.0-8.5). At the time of the study, 5 (8.3%) patients were dead, The causes of death included respiratory failure due to ILD progression (*n* = 3),advanced ovarian malignancy (*n* = 1), and sepsis (*n* = 1), mortality was considerably different between patients with and without ILD (*P* = 0.02). All patients in this study were on regular intravenous immunoglobulin (IVIg) replacement therapy. In the ILD group, 7 out of 10 patients (70%) received additional immunosuppressive treatment, including systemic corticosteroids (*n* = 5), azathioprine (*n* = 3), and mycophenolate mofetil (*n* = 2). The decision to initiate immunosuppressive therapy was based on the severity of ILD and the presence of other autoimmune or inflammatory complications.

More details are provided in Table 1.

**Table 1:** Demographic characteristics of CVID patients with and without ILD

Parameters	Total *(n = 60)*	With ILD *(n = 10)*	Without ILD *(n = 50)*	*P*-value
Sex ratio, M/F, (*n* = 60)	25/35 (41.6/58.4)	7/3	18/32	0.046*
Consanguinity, (*n* = 55) (%)	22 (40)	3 (42.9)	19 (39.6)	0.589
Family history of PID, (*n* = 60) (%)	5 (8.3)	1 (10)	4 (8.0)	0.612
Age, y, median (IQR)	18.5 (11.75-37.0)	27.0 (10.75–37.5)	17.0 (12.0–37.0)	0.576
Age at onset (year), median (IQR)	6.0 (2.75-11.0)	6.5 (2.37–16.25)	5.0 (2.5–11.0)	0.909
Age of diagnosis(year),, median (IQR)	11.0 (4.0–21.0)	14.0 (4.0–27.0)	11.0 (4.75–21.75)	0.843
Age of delayed diagnosis(year),, median (IQR)	3.9 (0.0-8.5)	3.8 (0.25–9.45)	4.0 (0.0-9.1)	0.980
Mortality, n (%)	5 (8.3)	4 (40%)	1 (2%)	0.02*

M, Male; F, Female; N, number; PID: Primary immunodeficiency diseases; ILD, interstitial lung disease; N/A, Not available

The median is shown [with 25th and 75th percentiles]

The p-value is statistically significant < 0.05

The most common clinical symptoms in CVID patients were cough, which was observed in 55% of patients, followed by pneumonia with 45.9%. Pneumonia was significantly different between the groups of patients with and without ILD (*P* = 0.002), and was observed in 100% of patients with ILD. The frequency of diarrhea in the patients with ILD was significantly higher than patients with ILD (*P* = 0.022). Hepatomegaly was more frequent in CVID patients with ILD (*P* = 0.029). Malignancy was observed in 6% of patients without ILD and was not significantly different from patients with ILD. Moreover, Autoimmunity was observed in 17 patients (28.3%) overall, with no significant difference between the ILD and non-ILD groups (30% vs. 28%, *p* = 0.586). Autoimmunity was defined as the presence of autoimmune cytopenias (immune thrombocytopenia, autoimmune hemolytic anemia, or autoimmune neutropenia) or other autoimmune diseases (e.g., autoimmune thyroiditis, inflammatory bowel disease, or rheumatoid arthritis) .In our study, immune cytopenia being the most frequent autoimmune manifestation. Immune thrombocytopenia was reported in 6 patients (10%), and autoimmune hemolytic anemia was observed in 4 patients (6.7%). Other clinical symptoms were not significantly different between the two groups (*P* > 0.05). Table 2 shows the clinical features of CVID patients with and without ILD.

**Table 2:** Clinical features of CVID patients with and without ILD

Parameters	Total *(n = 60)*	With ILD *(n = 10)*	Without ILD *(n = 50)*	*P*-value
**Respiratory Manifestations**				
Cough, n (%)	33 (55.0)	6 (60)	27 (54)	0.503
Pneumonia, n (%)	28 (45.9)	10(100)	18 (36)	0.002*
Sinusitis, n (%)	20 (33.3)	5 (50)	15 (30)	0.194
Otitis media, n (%)	17 (28.3)	4 (40)	13 (26)	0.295
Breath shortness, n (%)	10 (16.7)	1 (10)	9 (18)	0.468
Wheezing (*n* = 60) (%)	7 (11.7)	1 (10)	6 (12)	0.670
Bronchiectasis, n (%)	4 (6.7)	0 (0)	4 (8)	0.472
**Gastrointestinal Manifestations**				
Diarrhea, n (%)	22 (36.7)	7 (70)	15 (30)	0.022*
**Other Manifestations**				
Dermatologic disorders, n (%)	26 (43.3)	4 (40)	22 (44)	0.550
Autoimmunity, n (%) Immune cytopenia	17 (28.3) 10 (16.6)	3 (30) 2 (20)	14 (28) 8 ( 16)	0.586
FTT, n (%)	12 (20.0)	0 (0)	12 (24)	0.087
PND, n (%)	13 (21.7)	4 (40)	9 (18)	0.132
Splenomegaly, n (%)	13 (21.7)	4 (40)	9 (18)	0.132
Oral candidiasis, n (%)	10 (16.7)	1 (10)	9 (18)	0.469
Clubbing, n (%)	10 (16.7)	2 (20)	8 (16)	0.531
Fever, n (%)	9 (15.0)	1 (10)	8 (16)	0.533
Hepatomegaly, n (%)	5 (8.3)	3 (30)	2 (4)	0.029*
Abscess formation, n (%)	4 (6.7)	2 (20)	2 (4)	0.126
Cold, n (%)	3 (5.0)	1 (10)	2 (4)	0.427
Malignancy, n (%)	3 (5.0)	0 (0)	3 (6)	0.729

The median (IQR) of white blood cell (WBC) in patients with ILD trended higher in comparison with the patients without ILD [11.0 (7.3–15.6) × 103 (cell/uL) versus 6.75 (9.1–4.65) × 103 (cell/uL), *P* = 0.013]. There was a notable difference between platelets in the two groups of patients with and without ILD, and it was statistically higher in the group of patients with ILD (*P* = 0.011). The immunologic parameters reported in this study reflect the data at the time of CVID diagnosis, before the initiation of IVIg replacement therapy. The median IgG level was 581.0 mg/dL (IQR: 165.25–798.5), the median IgA level was 22.0 mg/dL (IQR: 9.0-69.5), and the median IgM level was 34.0 mg/dL (IQR: 17.0–68.0). According to Table 3, we found no significant differences in the other laboratory variables between the two groups (*P* > 0.05).

**Table 3:** Laboratory findings of CVID patients with and without ILD

Parameters	Total *(n = 60)*	With ILD *(n = 10)*	Without ILD *(n = 50)*	*P*-value
WBC × 10^3^ (cell/uL), median (IQR)	7.2 (5.1–9.4)	11.0 (7.3–15.6)	6.75 (9.1–4.65)	0.013*
Lymphocytes percent (%), median (IQR)	30.2 (24.0-43.2)	27.0 (22.5–38.5)	30.85 (24.0–45.0)	0.266
Neutrophils percent (%), median (IQR)	59.6 (47.7–66.2)	60.5 (54.0-66.25)	59.15 (46.17–66.75)	0.323
Hb (mg/dl), median (IQR)	12.0 (10.3–13.6)	10.9 (9.9–12.3)	12.25 (10.35–13.67)	0.243
Platelet × 10^3^ (cell/uL), median (IQR)	228.0 (154.25–324.5)	337.0 (242.0-494.0)	225.0 (136.5-279.5)	0.011*
CD3 + T cells %, median (IQR)	78.0 (63.0–84.0)	81.0 (76.0–83.0)	76.9 (62.25–84.75)	0.354
CD4 + T cells %, median (IQR)5	28.9 (21.0–37.0)	32.1 (21.75–48.5)	28.0 (20.0-35.5)	0.570
CD8^+^ T cells %, median (IQR)	36.5 (25.0–48.0)	37.0 (20.048.2)	36.25 (29.25-48.0)	0.742
CD16^+^ B cells %, median (IQR)	7.0 (5.4–12.0)	14.5 (8.0-18.15)	7.0 (5.3–11.0)	0.078
CD19^+^ B cells %, median (IQR)	7.7 (1.0-14.9)	3.6 (1.0–10.0)	8.0 (1.0–17.0)	0.231
IgG (mg/dL), median (IQR)	581.0 (165.25–798.5)	220.0 (96.75–419.5)	643.5 (193.75-839.25)	0.084
IgA (mg/dL), median (IQR)	22.0 (9.0-69.5)	16.0 (3.5–34.0)	26.5 (10.0-71.75)	0.163
IgM (mg/dL), median (IQR)	34.0 (17.0–68.0)	13.0 (5.0-42.5)	38.5 (19.0-73.25)	0.055
IgE (mg/dL), median (IQR)	2.8 (1.0–10.0)	1.0 (8.5–0.7)	4.0 (1.0-11.25)	0.386

Ig; Immunoglobulin, WBC; White blood cell; The median is shown [with 25th and 75th percentiles]; ILD, interstitial lung disease

* p-value is statistically significant < 0.05

Among the 60 patients, 29 patients had pulmonary involvement, and CT scan information was not available for 2 patients. Also, 29 patients had normal pulmonary CT scan. Nodule and peribranchial wall thickening were the most common radiological findings in patients (*n* = 8, 27.6%). After that, hyperinflation, atelectasis and consolidation were the most observed lung involvement (*n* = 7, 24.1%). There was no significant difference in pulmonary infiltration between patients with and without ILD (*P* = 0.326). A remarkable difference in terms of hyperinflation was observed between two groups, and the frequency was higher in the group of patients without ILD (*P* = 0.04). More details of radiological findings are given in Table 4.

**Table 4:** Pattern of lung involvement in CVID patients (*n* = 29) with and without ILD

Parameters	Total *(n = 29)* ^**^	With ILD *(n = 9)*	Without ILD *(n = 20)*	*p*-value^#^
Infiltration (*n* = 29) (%)	2 (6.9)	0 (0)	2 (10)	0.326
Hilar lymphadenopathy (*n* = 29) (%)	1 (3.4)	0 (0)	1 (5)	0.495
Hyperinflation (*n* = 29) (%)	7 (24.1)	0 (0)	7 (35)	0.040*
Atelectasis (*n* = 29) (%)	7 (24.1)	1 (11.1)	6 (30)	0.271
Nodule (*n* = 29) (%)	8 (27.6)	4 (44.4)	4 (20)	0.173
Fibrotic bond (*n* = 29) (%)	6 (20.7)	3 (33.3)	3 (15)	0.260
Consolidation (*n* = 29) (%)	7 (24.1)	4 (44.4)	3 (15)	0.086
Ground-glass opacity (*n* = 29) (%)	6 (20.7)	3 (33.3)	3 (15)	0.260
Interseptal Pleural thickening (*n* = 29) (%)	2 (6.9)	1 (11.1)	1 (5)	0.548
Peribronchial wall thickening (*n* = 29) (%)	8 (27.6)	1 (11.1)	7 (35)	0.183
Mosaic attenuation pattern (*n* = 29) (%)	3 (10.3)	1 (11.1)	2 (10)	0.928

ILD, interstitial lung disease

*The p-value is statistically significant < 0.05

**Among 60 included patients, 2 individuals had no lung CT-scan report, 29 patients had normal report and 29 patients had lung involvement.# p-value for comparison of patients with and without ILD.

Transbronchial biopsy (TBB) was performed in two patients with CVID-ILD. The histopathological findings revealed granulomatous inflammation and lymphocytic interstitial pneumonia(included CD3 and CD20 positive lymphocyte accumulations around the airway, some lymphocytes were also presented with BCL2.k167 nuclear staining).

, consistent with the diagnosis of ILD in these patients.

Pulmonary function tests (PFTs) were performed in 12 adult patients (6 with ILD and 6 without ILD). In the ILD group, the mean forced vital capacity (FVC) was 65.2% ± 12.3% of the predicted value, and the mean diffusing capacity for carbon monoxide (DLCO) was 58.7% ± 10.6% of the predicted value. In the non-ILD group, the mean FVC was 88.5% ± 8.2% of the predicted value, and the mean DLCO was 78.3% ± 7.9% of the predicted value. The differences in FVC and DLCO between the two groups were statistically significant (*p* < 0.05).

## Discussion

In the present study, we found that 16.6% of CVID patients had interstitial lung disease (ILD), which falls within the previously reported range of 10–20% in the literature [17–19]. However, it is important to note that the diagnosis of ILD in our cohort was primarily based on clinical and radiological findings, with only two patients undergoing transbronchial biopsy for histopathological confirmation. While radiological imaging is a crucial diagnostic tool, histopathological evaluation through lung biopsy remains the gold standard for definitive diagnosis and characterization of ILD subtypes [10, 16]. The limited availability of biopsy data in our study highlights the need for more comprehensive diagnostic approaches, including histopathological examination, to accurately assess the burden and patterns of ILD in CVID patients.

In the study by Lopes et al., 46 patients with biopsy-proven ILD were reported in 637 CVID patients (7.2%) [10]. In another study in Germany, among 105 adult patients with CVID, 19 patients also had ILD (18.09%). Cunningham et al. reported that the overall percentage of ILD in the CVID patients was 10.4% [21]. In our study, the incidence of ILD was reported as 16.6%. Similar to other reports in which the rate of ILD manifestations in CVID ranges from 10 to 20% [18, 22, 23]. In our study, the diagnosis of ILD was mostly based on radiological findings, while other reports focused on people who underwent biopsy to diagnose ILD [10].

Our findings revealed significant clinical associations between ILD and specific manifestations in CVID patients. Notably, we observed higher rates of pneumonia, diarrhea, and hepatomegaly in the ILD group compared to non-ILD patients. These findings align with previous reports suggesting that CVID-ILD patients often exhibit a distinct clinical phenotype characterized by increased inflammatory and autoimmune complications [14, 15]. The higher incidence of pneumonia in ILD patients may be related to the underlying lung pathology, while diarrhea and hepatomegaly could be manifestations of the systemic immune dysregulation associated with CVID-ILD .

Regarding laboratory parameters, our study found significantly higher platelet counts and white blood cell counts in the ILD group compared to non-ILD patients. These findings may reflect the persistent inflammatory state and immune dysregulation observed in CVID-ILD patients [10, 18]. However, further research is needed to elucidate the mechanistic links between these laboratory abnormalities and the pathogenesis of ILD in CVID. Kellner et al., who studied the cellular defects of CVID patients with chronic lung diseases, showed that CVID patients with ILD had lower counts of CD3, CD4, and CD8 cells compared to the group without this type of lung involvement. While in our study, CD3, CD4 and CD8 cells were not significantly different between the two groups of patients with and without ILD. The different sample size may be the cause of this discrepancy [18].

Infections and bronchiectasis are primarily due to antibody deficiency, but interstitial lung disease associated with CVID-ILD is best considered part of a systemic immune dysregulation process, with CVID-ILD individuals often having splenomegaly, lymphadenopathy and are autoimmune [17, 24]. In this regard, in the current study, the rate of hepatomegaly was significantly higher in ILD patients.

According to previous studies, ILD patients usually have more inflammatory complications and clinical manifestations than non-ILD patients. Kellner et al., reported that pneumonia, herpes virus and fungal infection in ILD patients was higher than non-ILD patients [18]. In the study by Lopes et al., splenomegaly and lymphadenopathy were present in approximately two-thirds of ILD patients, and more than half had cytopenias [10]. In our study, the rate of pneumonia and diarrhea in ILD patients was significantly higher than in non-ILD patients. A high frequency of splenomegaly and a history of cytopenia have also been highlighted as potential predictors of granulomatous lymphocytic interstitial lung disease (GLILD) by Hartono et al [24].

In our study, we observed a higher frequency of hyperinflation on CT scans in non-ILD patients compared to those with ILD. This finding contrasts with previous reports suggesting an association between hyperinflation and ILD in CVID patients. It is possible that the radiological patterns in our cohort were influenced by the predominant reliance on clinical and radiological criteria for ILD diagnosis, rather than histopathological confirmation. Additionally, the small sample size may have contributed to this discrepancy. Nonetheless, our findings underscore the heterogeneity in radiological manifestations of ILD in CVID patients and highlight the importance of integrating radiological data with clinical, laboratory, and histopathological findings for accurate disease characterization.

We found that bronchial wall thickening was not significantly different between patients with and without ILD. Also, the nodule and fibrotic band were statistically similar between the two groups.

Fraz et al., reported several ILD patients had traction bronchiectasis, a common finding in ILD, and this finding was significantly more prevalent in patients with clinically advanced Granulomatous-Lymphocytic interstitial lung disease (GLILD). Furthermore, interlobular septal thickening and traction bronchiectasis were clearly different between subjects with and without clinical progression. Faint central lobular nodules and small subpleural nodules were unusual findings in patients [25]. In the present study, no meaningful difference was observed between patients with and without ILD in terms of nodules and interseptal pleural thickening. The limitations of the study were the small sample size, lack of genetic examination of patients, and lack of biopsy to diagnose ILD for all patients.

One of the most striking findings of our study was the significantly higher mortality rate observed in CVID patients with ILD compared to those without ILD. This result highlights the substantial impact of ILD on the prognosis and survival of CVID patients. The main causes of death in the ILD group were respiratory failure secondary to ILD progression (*n* = 3), advanced ovarian cancer (*n* = 1), and sepsis (*n* = 1). These findings underscore the need for close monitoring, early diagnosis, and prompt treatment of ILD in CVID patients to improve outcomes and reduce mortality. Our results are consistent with previous studies reporting increased morbidity and mortality in CVID patients with ILD [11, 26]. The higher mortality observed in our ILD cohort underscores the substantial burden and prognostic implications of this complication in CVID patients. These findings emphasize the need for close monitoring and multidisciplinary management strategies for CVID-ILD patients, who represent a high-risk subgroup with increased morbidity and mortality.

Our study had several limitations, including a relatively small sample size and the lack of genetic examination or histopathological confirmation of ILD for all patients. Future studies should aim to incorporate comprehensive genetic analyses to elucidate the potential genetic factors contributing to the development of ILD in CVID patients. Additionally, larger multicenter studies with standardized diagnostic protocols, including lung biopsies, are warranted to further characterize the diverse clinical, radiological, and histopathological patterns of ILD in CVID patients.

Given the complex interplay between ILD and CVID, a deeper understanding of the underlying immunopathogenesis is crucial for developing targeted therapeutic strategies and improving patient outcomes. Future research should focus on elucidating the molecular mechanisms and immune dysregulation pathways that contribute to the development and progression of ILD in CVID patients. Such insights may pave the way for personalized treatment approaches and enhance our overall understanding of this challenging complication.

## Conclusion

In conclusion, we showed that pneumonia, diarrhea, otitis, and hepatomegaly were statistically higher in the group of patients with ILD. Als, from radiological findings, the frequency of, hyperinflation was higher in the non- ILD patients. Respiratory complications actually determine the prognosis of the disease. It is necessary to expand our understanding of the etiology and immunopathogenesis of ILD in CVID in order to provide a more accurate prognosis and select appropriate treatments. Due to the complex course of these disorders, regular examinations and close monitoring of these vulnerable patients using multidisciplinary healthcare is strongly recommended.

## Data Availability

The datasets generated and analyzed during the current study are not publicly available due to limitations of ethical approval involving the patient data and anonymity but are available from the corresponding author on reasonable request.
